# Improving parameter recovery for conflict drift-diffusion models

**DOI:** 10.3758/s13428-020-01366-8

**Published:** 2020-02-10

**Authors:** Ronald Hübner, Thomas Pelzer

**Affiliations:** Department of Psychology, Universität Konstanz, D-78457 Konstanz, Germany

**Keywords:** Drift-diffusion models, Parameter recovery, Model-fit procedure, Grid-search method

## Abstract

Several drift-diffusion models have been developed to account for the performance in conflict tasks. Although a common characteristic of these models is that the drift rate changes within a trial, their architecture is rather different. Comparative studies usually examine which model fits the data best. However, a good fit does not guarantee good parameter recovery, which is a necessary condition for a valid interpretation of any fit. A recent simulation study revealed that recovery performance varies largely between models and individual parameters. Moreover, recovery was generally not very impressive. Therefore, the aim of the present study was to introduce and test an improved fit procedure. It is based on a grid search for determining the initial parameter values and on a specific criterion for assessing the goodness of fit. Simulations show that not only the fit performance but also parameter recovery improved substantially by applying this procedure, compared to the standard one. The improvement was largest for the most complex model.

## Introduction

The ability to act in a goal-oriented manner is an essential characteristic of human performance. To investigate involved mental processes, several so-called *conflict paradigms* have been developed, such as the Stroop task (Steinhauser & Hübner, 2009; Stroop, 1935), the Eriksen flanker task (Eriksen & Eriksen, 1974), and the Simon task (Hübner & Mishra, 2013; Proctor, 2011; Simon, 1969), where irrelevant stimulus features produce response conflicts that are reflected by *congruency effects.* Recently, conflict DDMs (drift diffusion models) have been proposed that, based on response-time (RT) distributions and accuracy data, model the dynamics of the performance in conflict tasks. The first of these models was the Dual-Stage Two-Phase (DSTP) model (Hübner, Steinhauser, & Lehle, 2010), followed by the Shrinking Spotlight (SSP) model (White, Ratcliff, & Starns, 2011). Both models were first applied to the flanker task. Later, the Diffusion Model for Conflict (DMC) tasks (Ulrich, Schröter, Leuthold, & Birngruber, 2015) has been proposed, which has also been applied to Simon-task data. If interpreted accordingly, however, the DSTP model can be used as well to model Simon-task data (Hübner & Töbel, 2019).

In studies, in which conflict tasks are modeled, one or several of the considered models are fitted to experimental data in the same way as common DDMs, and much effort is usually spent to obtain good fits. However, although a good fit is important for modeling, it does not guarantee that the obtained parameters are valid (Pitt & Myung, 2002; Roberts & Pashler, 2000). Indeed, it is possible that a model fits data satisfactorily, but the corresponding parameter values are rather different from those in the underlying population. In this case the model and/or fitting procedure did not validly recover the true parameter values of the population. It can therefore be argued that data recovery is just as important, if not more important, for modelling than a good fit, especially if a model is fitted to individual data and the obtained parameter values are used for diagnostic purposes.

Because parameter recovery is an important issue, several studies have tried to assessed the corresponding performance of DDMs (e.g., Lerche & Voss, 2016; van Ravenzwaaij & Oberauer, 2009). For conflict DDMs, however, recovery performance has not been investigated until recently, when White, Servant, and Logan (2018) simulated flanker-task data. In the flanker task (Eriksen & Eriksen, 1974), a central target item, which has to be categorized, is presented along with two or more irrelevant flanker items. A stimulus is said to be congruent or incongruent, depending on whether the flankers activate the correct or wrong response, respectively. Thus, in contrast to congruent stimuli, incongruent stimuli produce a response conflict, which is usually expressed by an increased RT and error rate. White et al. (2018) found that recovery performance largely differed between the three conflict models and individual parameters. Remarkably, their results have also been interpreted as indicating a generally poor parameter recovery of the three conflict DDMs, which led to their exclusion from modeling (Weigard, Heathcote, & Sripada, 2019). This example shows how important a good recovery performance is for model selection. Because parameter recovery not only depends on the model as such but also on the applied fit procedure, the aim of the present study was to investigate to what extent recovery performance of conflict DDMs can be improved by using a fit procedure that is more sophisticated than those usually applied.

### Parameter estimation

In formal modeling, it is assumed that the performance of a person or population can be represented by a set of model parameters. The specific values of these parameters depend on the persons and on the condition in which the behavior is observed. These parameter values are estimated by fitting the model to observed data. The estimation involves the usual problems concerning the properties of estimators, such as *consistency* and *efficiency*. Unfortunately, up to now, little is known about these properties with respect to DDMs.

As mentioned, the basis of parameter estimation is observed data. In applications of DDMs, the considered data are usually RTs and proportions of correct and wrong responses, respectively. For simple versions of DDMs, for which analytic expressions of the densities exist, maximum-likelihood procedures can be used to estimate the parameter values. However, for most of the usually applied models no analytic expressions are available. Therefore, model data are simulated and compared to the observed ones. For this objective, the observed RTs are usually summarized by quantiles of the corresponding cumulative distribution functions (CDFs). Often, five quantiles (0.1, 0.3, 0.5, 0.7, 0.9) are computed for correct and incorrect responses, respectively. In conflict tasks, however, categorization accuracy is often near perfect in the *congruent* condition, and thus very few (possibly zero) observations are available to estimate the quantiles of the error response time distribution with. Few errors might be summarized by a single quantile (e.g., the median). Often, however, especially if one models individual data, there are no errors at all. Therefore, Hübner (2014) suggested to represent errors by corresponding proportions derived from so-called *conditional accuracy functions* (CAFs, De Jong, Liang, & Lauber, 1994). A CAF is usually constructed by first dividing the distribution of *all* RTs (correct and error RTs) by means of quintiles (i.e., .2, .4, .6, .8) into five equal sized intervals: (0, .2), (.2, .4), (.4, .6), (.6, .8), (.8, 1). Accuracy (i.e., correct responses divided by all responses) in each interval is then plotted against the mean RT in the corresponding interval. CAFs are an informative data representation, because they nicely visualize how accuracy varies with RT. Whereas accuracy for congruent stimuli has a constantly high level across RT, that for incongruent stimuli is usually low for fast responses, but increases with RT. In any case, error proportions can easily be computed from CAFs if few or even no errors are present in the congruent condition (for details see below).

The summary data span a multidimensional space, whose dimensions depend on the number of considered CDF and CAF quantiles, respectively. The parameters of a model also span a multidimensional space. Accordingly, each set of parameter values defines a point ***p*** in this parameter space ***P***. A model can therefore be considered as function *m* that maps a point in parameter space to a point ***r*** in data space ***R*** :

1 $$ m\left(\boldsymbol{p}\right)=\boldsymbol{r}. $$

Because of the missing analytical formulas for *m*, the function must be approximated by a procedure, usually the Euler–Maruyama schema (Maruyama, 1955), which approximates the numerical solution of the corresponding stochastic differential equation by means of a Wiener process. Consequently, each approximation produces a slightly different value, i.e., set of data (given the random-number generator starts with a different seed). As a result, *m* now maps one ***p*** to multiple ***r*** and, therefore, represents a multivalued function. The simulated result can be expressed by means of a multi-dimensional error term ***e*** and the true ***r*** (see Eq. 1), i.e., we have:

2 $$ m\left(\boldsymbol{p}\right)=\boldsymbol{r}+\boldsymbol{e} $$

Although the exact distribution of Eq. 2 is not known, it is reasonable to assume that, due to characteristics of the Wiener process, ***e*** is normally distributed. Moreover, with an increasing number *n* of simulations the variance of the error term decreases so that ***e*** approaches zero. This means that the solution of Eq. 2 relates to more or less points in data space depending on *n*. The smaller *n*, the larger the number of points, and, consequently, the larger is the possibility of an overlap of points resulting from different parameter sets. Unfortunately, for the models considered here, little is known in this respect.

Given observed distributional data (quantiles, proportions), one task of modeling is to find parameter values that produce the most similar data. The goodness of fit is assessed by considering a corresponding measure such as chi^2^ or *RMS* (root mean square) error. The smaller the corresponding values the better is the fit. Each of these measures defines a scalar evaluation function *f* that maps a given point ***r*** in data space, and a point ***p*** in parameter space to a single value *g*, reflecting the goodness of fit. It should be noted that, even though *f* takes ***p*** as an argument, it computes the goodness of fit by means of *m*. This reduces *f* to a mere comparator function between two data sets:

3 $$ f\left(\boldsymbol{p},\boldsymbol{r}\right)\equiv f\left(m\left(\boldsymbol{p}\right),\boldsymbol{r}\right)=g. $$

Thus, modeling consists of searching for a set of parameter values ***p*** that minimizes *g*. Finding the minimum of the evaluation function is usually achieved by applying minimization algorithms such as the Simplex (Nelder & Mead, 1965) or Powell’s (Brent, 1973) algorithm. They systematically vary parameter values in such a way that the deviation between observed and simulated data, i.e., the goodness-of-fit value *g*, is gradually reduced until a minimum is found. The finally obtained parameter values are then taken as estimates of the respective population values.

### Parameter recovery

As mentioned, even if a set of parameter values is found that produces a good fit to the data, this does not guarantee that the parameters are close to the parameters of the population. This is the parameter-recovery problem. There are several possible reasons for a bad recovery, most of which have to do with properties of the solution space of the function given by Eq. 2. One possibility is that the fit routine gets trapped in a *local* minimum and therefore does not find the *global* minimum of the evaluation function. This problem is usually addressed by starting the fit process several times beginning from different starting points, which increases the probability that the global minimum is found. Another measure is to restrict the considered parameter space. If the region of plausible parameter values is known, then one can impose restrictions, which might reduce the number of local minima.

Furthermore, a reliable recovery is possible only if there is a functional one-to-one mapping between parameter space and data space. If several sets of parameter values produce the same data, then it is impossible to uniquely decide whether the estimated values reflect the original ones or not. It can also be the case that different sets of parameters produce the same goodness-of-fit with different data. For instance, one set leads to a better fit of the error RTs, whereas another produces a better fit to the correct RTs. If different parameter sets produce the same goodness of fit, one could choose the values that are more plausible.

Additionally, because *f* takes *m*(***p***) as argument, there will also be a variability in *g* itself, even if the same ***p*** is used:

4 $$ f\left(m\left(\boldsymbol{p}\right),\boldsymbol{r}\right)=f\left({\boldsymbol{r}}_m+{\boldsymbol{e}}_m,\boldsymbol{r}\right)=g+e. $$

Moreover, it is usually assumed that the observed data ***r*** themselves are noisy, which further reduces the reliability of *g*.

The properties of the parameter space depend on the model. For instance, a model can be over-parameterized, or the parameters are strongly related. The risk that this is the case increases with the number of parameters. The uniqueness of the mapping from parameter space to data space, however, also depends on the representation of the data space. The smaller the number of dimensions, the more likely it is that different parameter sets produce the same data or goodness of fit. An extreme case is given, for instance, if only the means or medians of the data are considered. Therefore, it is a great advantage of DDMs, that they can also model multiple distributional data. But even in this case it is likely that the mappings are not unique.

In practice, the true population parameter values are unknown. However, to assess the models and fit procedures with respect to recovery performance, one can use the models and simulate data with a given set of parameter values. If the model recovers well, the fit procedure should always find values that are close to the original ones. This is the method followed by White, Servant, and Logan (2018) in their study of the three conflict-task models.

As mentioned, recovery performance depends on the model as well as on the fit procedure. Therefore, if a model does not recover well, the exact reason remains open. Because White et al. (2018) kept the fit procedure constant, they were able to compare model performance. However, it is possible that models and fit procedures interact. The present study demonstrates that this is indeed the case. The most complex model profits more from an improved fit procedure than the other models. In other words, optimizing parameter recovery can require a more sophisticated fit method for complex models, compared to simple ones.

## Models

All three considered models are based on a response-selection mechanism, implemented as diffusion process (cf. Ratcliff, 1978). This process is characterized by a drift rate *μ* reflecting the evidence available for response A relative to response B and by two corresponding thresholds *A* and –*B*. Responses A and B usually represent a correct and a wrong response, respectively. Noisy samples of the evidence are accumulated beginning at *t*_*0*_ with value *X*_*0*_, until threshold *A* or –*B* is reached. The duration of this process is the decision time. It is assumed that the response time is the sum of this decision time and some non-decisional time *t*_*er*_, representing the duration of processes such as stimulus encoding, response execution, etc. The complexity of the diffusion process can further be increased by assuming that the starting value, the non-decisional time, and/or the rate vary randomly across trials according to specific distributions (Ratcliff & Rouder, 1998). In the original DMC application, Ulrich et al. (2015) assumed across-trial variability for the starting value and the non-decision time. For simplicity, White et al. (2018) dropped these assumptions. In the present study, we made the same simplifications.

Whereas all models assume such a single response-selection process, the models largely differ in their architecture. A helpful visualization of the three architectures is provided by White et al. (2018, Fig. 1). Here, we limit their description to a short introduction.

**Fig. 1:**
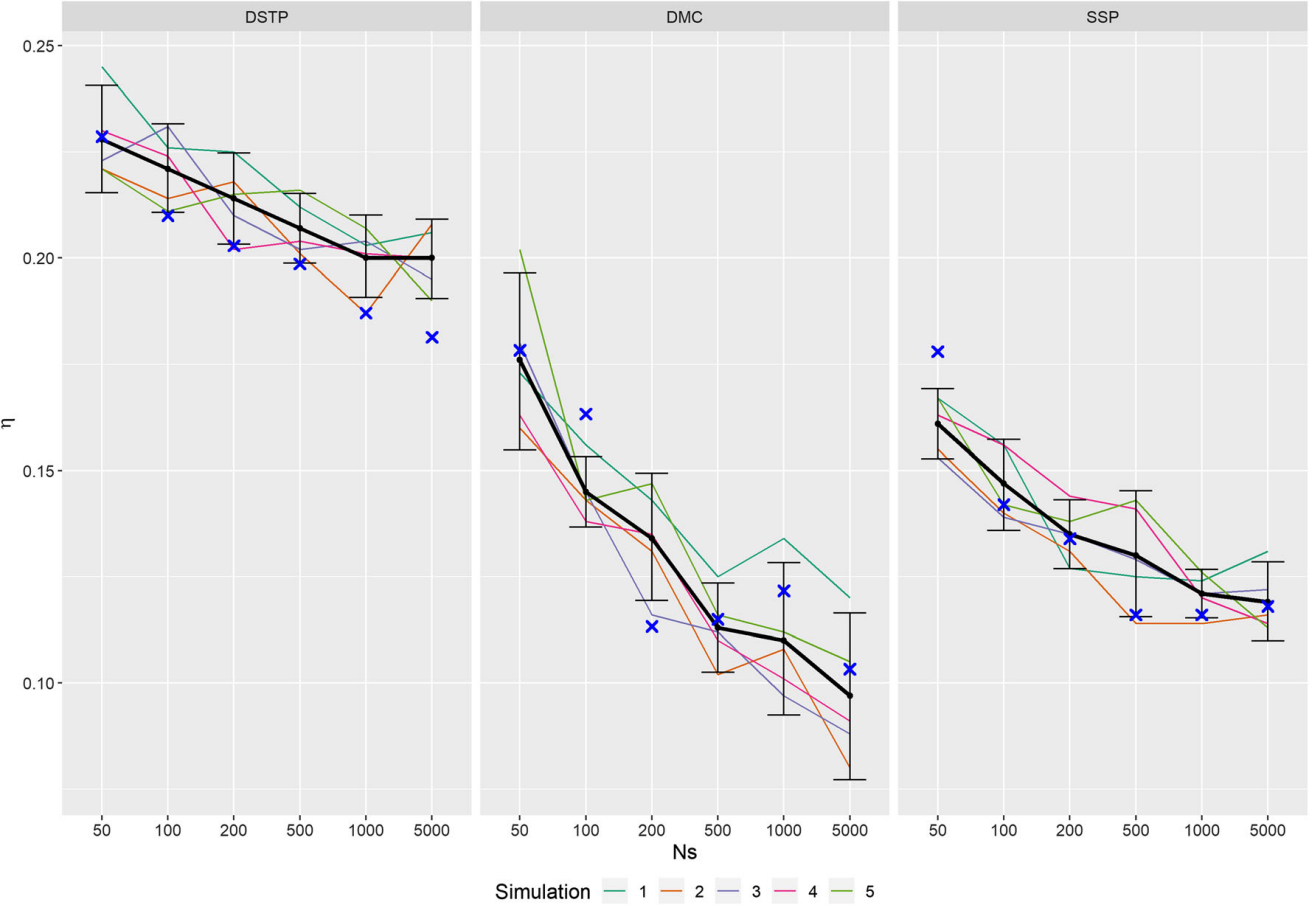
Recovery performance for the three models in the situation that was similar to that in White et al. (2018). The *colored lines* show the results for the five simulations. The *black thick line* is the corresponding mean performance. The *errors bars* are constructed based on variability across the five runs and indicate the 95% confidence interval. The *blue crosses* represent the results from White et al. (2018)

### SSP model

In the SSP model the overall rate for a given stimulus is computed from the weighted evidence provided by the target and flankers. It is assumed that all items provide the same amount of perceptual evidence *p*. However, the weight for each item is determined by the proportion of the attentional ‘spotlight’ that falls on the item’s location in the display. Selectivity, and consequently the influence of the target stimuli on the drift rate, increases gradually as the width of the target-centered spotlight shrinks over time at a linear rate, *r*_*d*_, from *sd*_*0*_ to a minimum. Thus, if we assume that *A* = *B*, the model has, including *t*_*er*_, five parameters.

### The DMC model

The specific idea for the DMC model (Ulrich et al., 2015) is based on the dual-route model (De Jong et al., 1994; Kornblum, Hasbroucq, & Osman, 1990), which assumes that task-relevant and task-irrelevant activations result from a controlled and from an automatic process, respectively, and that these activations are transmitted through separate, parallel processing pathways. Moreover, whereas the rate *μ*_*c*_, representing the controlled process, remains constant, the rate *μ*_*a*_(*t*) resulting from the automatic processes varies as a function of time *t*. The dynamics of this rate is modeled by a gamma density function with shape parameter *a* > 1 and scale parameter*τ*. The function is further scaled (multiplied) by a parameter *m* reflecting the strength of automatic activation.

The DMC model further assumes that the relevant and irrelevant activations superimpose. This means that the overall drift rate at time *t* is the sum of the rate for the controlled process and the rate at time *t* for the automatic process. Furthermore, it is assumed that the rate of the automatic process is of the same size for congruent and incongruent stimuli, but that its sign is negative for incongruent stimuli, which can easily be achieved by scaling the rate with *–m* instead of *m*. Thus, the overall rate for selecting a response to a congruent stimulus is *μ*(*t*) = *mμ*_*a*_(*t*) + *μ*_*c*_. Altogether, the number of parameters for the DMC model adds up to six.

### The DSTP model

The main characteristics of the DSTP model are two discrete stages of information selection, an early, and a late stage determining the rate of response selection. Response selection starts with the rate of evidence provided by Stage 1. This stage is already selective, for instance by applying perceptual (e.g., spatial) filters, although selectivity is far from perfect. It is assumed that the rate *μ*_*RS1*_ for the first phase of response selection is composed of two component rates, *μ*_*t*_, and *μ*_*f*_, which are the result of the early stage of stimulus selection. The components represent the evidence provided by the target and the flankers in favor of the correct response *A*, respectively. Both components sum up to the total rate, i.e., *μ*_*RS1*_ = *μ*_*t*_ + *μ*_*f*_. The component *μ*_*f*_ is positive, if the flankers are response compatible, but negative, if they are incompatible. Thus, the rate *μ*_*RS1*_ is usually smaller for incongruent than for congruent stimuli, and can even be negative.

Additionally, a second and more effective stage of stimulus selection is assumed. If the processes at this stage finishes, the rate for response selection usually changes, which divides response selection into a first and a second phase (Phase 1 and Phase 2). Information selection at Stage 2 is also modeled by a diffusion process, running in parallel with response selection during Phase 1. If the evidence accumulated by this process in favor of some information C relative to information D hits threshold *C* or –*D*, then the rate of response selection changes to a corresponding value. However, it can also happen that a response is already selected during Phase 1.

To account for the fact that accuracy for incongruent stimuli usually improves with RT, the diffusion process that initiates a rate change of response selection is assumed to represent a late categorical stimulus-selection process SS with rate *μ*_*SS*_. It selects the mental category of the target letter or that of the flanker letter, depending on whether it hits thresholds *C* or –*D*, respectively. If the target is selected, then response selection continues with rate *μ*_*RS2C*_, which is usually higher, compared to *μ*_*RS1*_. In case the flanker was selected, the new rate is *μ*_*RS2D*_. This rate is positive or negative depending on whether the flanker is congruent or incongruent, respectively.

For the model applied in this study, we assumed symmetric thresholds for response and stimulus selection, i.e., *A*=*B*, and *C*=*D*. Furthermore, we assumed that target and flanker letter selection leads to the same rate for response selection in Phase 2, i.e., *μ*_*RS2C*_ = *μ*_*RS2D*_. Thus, altogether, the model has seven parameters.

## White et al.’s Study

In their recovery study, White et al. (2018) simulated flanker-task data for each of the three models. For each model, they used *N*_*pop*_ = 100 parameter sets, each representing a population. The corresponding values were randomly drawn from uniform distributions, whose ranges were determined by values found in empirical studies. Each parameter set was used to simulate data of six different sample sizes *N*_*S*_ (50, 100, 200, 500, 1000, and 5000). Each sample comprised a congruent and an incongruent condition. Thus, altogether, they simulated 600 data samples of different sizes. They then fitted the three models to these data samples with different fit procedures.

### Fit procedure

The simulated data samples were summarized by distributional data. Correct RTs were represented by five quantiles (0.1, 0.3, 0.5, 0.7, 0.9) in each condition. For error RTs, whose number is often very small, the quantiles were determined by an adaptive median-based procedure. More specifically, the five quantiles were computed only if there were more than ten errors. If the number of errors was ten or smaller but larger than five, three quantiles (.3, .5, .9) were computed. Five or fewer errors were represented by their median. Alternatively, they also applied a method proposed by Hübner (2014) that extracts error proportions from CAFs.

As goodness-of-fit measure, they used chi^2^:

5 $$ {\chi}^2=\sum \limits_{i=1}^2{N}_i\sum \limits_{j=1}^X\frac{{\left({o}_{ij}-{\pi}_{ij}\right)}^2}{\pi_{ij}}, $$

where *N*_*i*_ is the number of observations in compatibility condition *i*. The quantities *o*_*ij*_ and *π*_*ij*_ are the observed and predicted proportion *j* in condition *i*, respectively. As can be seen, the deviation of each point is weighted by the proportion of the corresponding data.

The Simplex algorithm (Nelder & Mead, 1965) was applied to find parameter values that minimize chi^2^. To reduce the risk that the result represents a local minimum, a two-step procedure was used. In the first step 20 fit processes were run, each with a different randomly chosen set of starting values, drawn in the same way as the parameter sets for producing the data samples. 10,000 trials were simulated in each compatibility condition at iteration of the parameter optimization algorithm. In the second step the parameter sets resulting from the two best fitting processes were used to continue the fit process with 50,000 trials per condition and fit cycle. The best fit was then considered as final solution. This two-step procedure was applied to all 600 data samples of each model.

### Assessment

The recovery performance of the models was assessed by comparing the original parameter values with the estimated ones. As goodness-of-recovery measure for a given parameter θ_*i*_ and sample size they computed the quantity *η*_*i*_:

$$ {\eta}_i=\sum \limits_{j=1}^{N_{pop}}\frac{\left(\left|{simulated\theta}_{ij}- recovered{\theta}_{ij}\right|\right)}{range{\theta}_{ij}}, $$

where the summation extends over the *N*_*pop*_ = 100 populations.

As a result, White et al. found that, overall, recovery was relatively good for all models. However, different parameters recovered differently well. Recovery performance also improved generally with sample size.

For comparing model performance, they summed *η*_*i*_ over the parameters of each model. We think that summation is not appropriate, because it complicates a direct comparison between models that differ in their parametrization, which is the case for the three models under consideration. Even though the result, that the SSP model recovered best and the DSTP model worst, still holds if model parameterization is taken into account, the difference between the models is reduced.

Concerning the method for summarizing error data, the median-based procedure was slightly better than the CAF-based one. Summed over all sample sizes, models, and parameters, the first method results in *η =* 1670, whereas the second gives *η =* 1725. If we consider the corresponding means, however, then that of the first procedure is 15.46 and that of the second procedure 15.97, which is a relatively small difference.

## Our study

A first goal of our study was to replicate White et al.’s (2018) results. For this objective we simulated data and fitted the three models in the same way as these researchers. In addition, we repeated all simulations four times to assess the variability of data simulation and model-fitting procedures.

The main goal of our study, however, was to compare different fit methods and their relation to parameter recovery. As already mentioned, a problem in model fitting is that minimization algorithms often get trapped in local minima, which can lead to a poor goodness of fit and parameter recovery. A common method to address this problem is to run the fit process several times, each with a different set of initial parameter values. White et al. (2018), for instance, used 20 randomly selected sets of start values for each of their model fits. Here, we sought to investigate whether this part of the fitting procedure could be improved by the addition of a grid search method (Zielesny, 2011) prior to the two-step procedure of White et al. (2018).

### Method

Our computational method was very similar to that used by White et al. (2018). We integrated, after translation, crucial parts of their Python program into our C++ program. As in White et al., the performance of all models was approximated by the Euler–Maruyama method (Kloeden & Platen, 1992). Integration constants and diffusion coefficients were the same as in White et al. We also applied the Simplex algorithm (Nelder & Mead, 1965) for minimization.

### Data simulation

We randomly selected *N*_*pop*_ = 100 sets of parameter values for each model in the same way as White et al. (2018). These parameter sets were then used to simulated data samples of sizes *N*_*S*_ = 50, 100, 200, 500, 1000, and 5000. Each data set represents the performance in a conflict task with a congruent and an incongruent condition. Different from White et al., we additionally repeated the basic simulations four times. For each repetition, 100 new sets of parameter values were randomly selected. Thus, altogether, we had *N*_*pop*_= 500.

### Model fitting

#### Goodness-of-fit measures

As summary statistics for the data samples, we used five CDF quantiles (.1, .3, .5, .7, .9) for correct responses and the accuracies in the five CAF intervals resulting from quintiles. These 10 values were computed for congruent as well as for incongruent trials. Thus, each data sample was represented by 20 values (*o*_*ij*_). The RTs corresponding to these values were then used as cut points in the predicted (simulated) CDF and CAF, respectively (because a CAF cannot be expressed on a per response basis, we computed the conditional accuracy for every percentile. If a cut point fell in between two percentiles, we calculated the corresponding accuracy by linear interpolation between the two adjacent percentiles). The quantiles (CDF) and accuracies (CAF) found using these cut points were then used as values *π*_*ij*_ for comparison with the corresponding values of the data sample (see. Eq. 5).

In addition to the chi^2^-based method, we also applied another method. Instead of comparing quantiles and accuracies corresponding to certain RT cut points, we compared RTs and accuracies corresponding to quantile/interval cut points. That is, we simply used the same summary statistics for the predicted data that were used for the data samples. This increased the number of values for comparison from 10 to 15 (five CDF RTs, five accuracies, and five mean RTs of the CAF intervals).

One characteristic of this method is that we have to deal with different units of measurement, i.e., millisecond and proportion. To treat the respective values equally, we used the *squared percentage error* (SPE) between observed and predicted values as fit criterion:

6 $$ SPE=\sum {\left(\frac{o_i-{s}_i}{o_i}\right)}^2, $$

where *o*_i_ is a quantity from the population sample, and *s*_i_ the corresponding quantity obtained by the fitting process. Because each component of the SPE represents the relative deviation in percentage, measures of different units are mapped to a common scale. Accordingly, they are weighted equally, which would not be possible with the chi^2^ criterion.

However, the deviation sensitivity of the SPE depends on the size of the respective value. The larger the observed value, the greater the deviation of the simulated data must be to produce a similar change in SPE. This characteristic produces a bias, because similar absolute deviations from small values have a larger effect on the SPE than those from larger values. However, because the bias remains constant for a given empirical data sample, comparisons between different parameterizations or *n* regarding a fixed data sample are still valid.

**Fig. 2:**
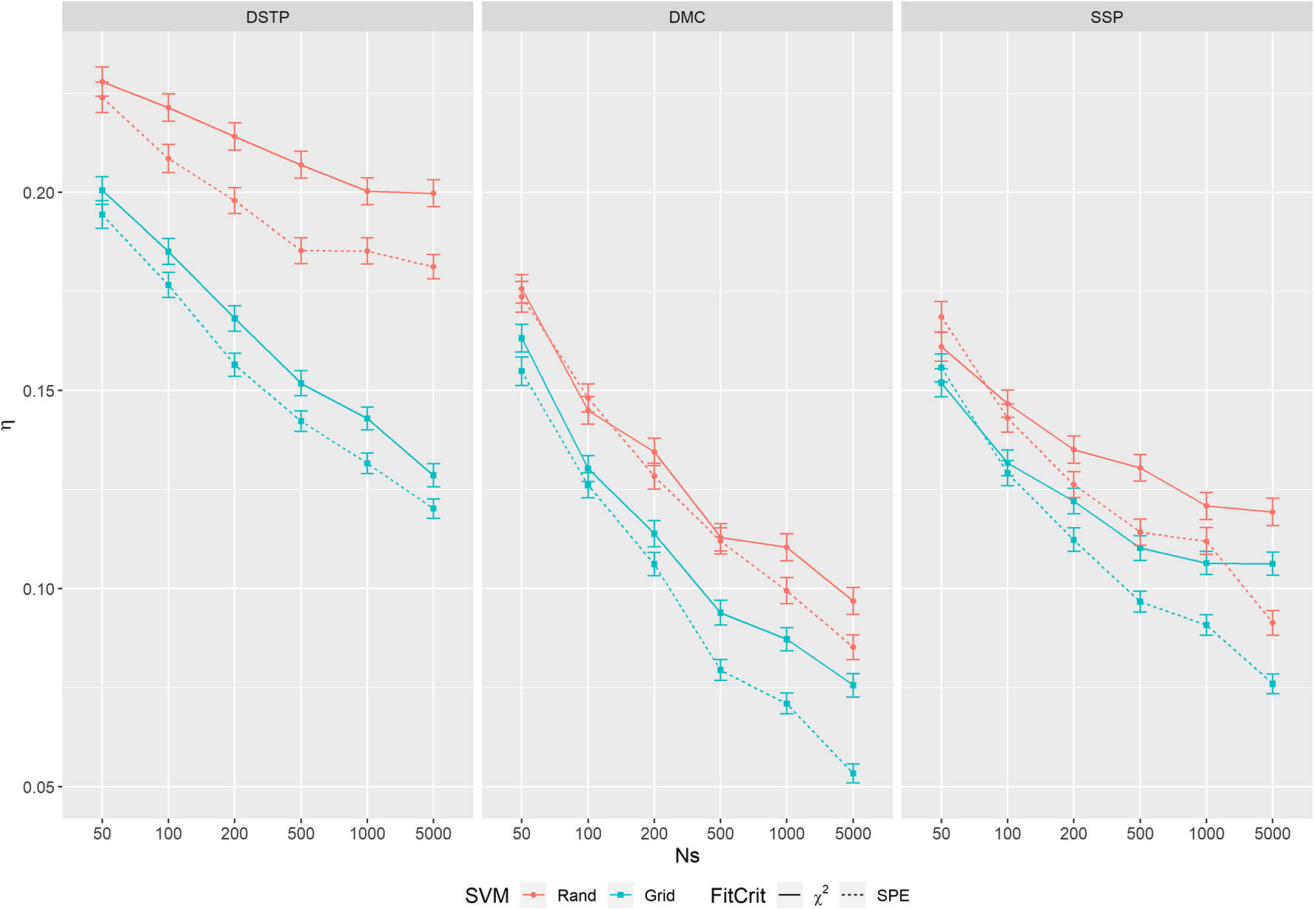
Mean η for all models, *N*_*S*_*,* fit criteria, and start methods. The goodness-of-recovery measures were averaged across all *N*_*pop*_. The *error bars* represent the 95% confidence interval

Nevertheless, with respect to comparing SPEs, we have to distinguish between the situation, where different data samples are fitted by the same model, and the situation, where one data sample is fitted by different models. Whereas in the former situation differences can only be interpreted if the observed samples are similar, this is not necessary in the latter situation. Because different models are fitted to different data samples in the present study, as in White et. al., and the considered models show different limitations regarding RTs (see Fig. 2 in White et. al.), we decided not to compare obtained SPE values between the three models.

#### Starting values

For the replication part, we used the same two-step procedure as White et al. (2018), which was described above and will be called the *rand* method. In addition to the rand method, however, we also applied a *grid-search* method (Zielesny, 2011). Instead of relying on a random selection of 20 start values, we systematically sampled the whole parameter space to find 20 starting values that already produced relatively good fits. For this objective, we defined for each parameter *N*_*g*_ equally spaced grid points inside its value range. The points of all parameters constitute a multidimensional grid, i.e. a systematic subset of the whole parameter space. We defined the grid size by the number of points per parameter, i.e. by *N*_*g*_. After some preliminary simulations, we came up with a grid size of six as appropriate for our objective. For a model with *n* parameters and a grid size of *N*_*g*_, the grid includes (*N*_*g*_)^*n*^ sets of parameter values. For instance, if a model has six parameters, then the grid includes 46,656 sets of parameter values.

Grid search means to simulate some trials for each point (parameter set) in the grid and to compare the result to the sampled data. Because to repeat this procedure for each sample size would have taken rather long, we computed the trial data only once for each point in the grid and stored the results.

It is important to note that the limitations with respect to the validity of data representation mentioned in the Introduction (see Eq. 2) also hold for the generated grid. This means that the error strongly depends on the number of simulated trials, which was 10,000 in the present case (all simulations started with a random seed). The required resources for the grid-search method are also dependent on the used fit criterion. For computing chi^2^ the complete RT distributions must be stored. This is necessary for finding the cut points corresponding to the RTs in the summary distributions of the data sample, which are needed for computing the respective proportions. This requires large amounts of storage space and later also much time for reloading. For instance, the stored distributions for the DSTP model alone had a size of 89 GB. However, this value strongly depends on the resolution and range of the data distribution. In our case, we conservatively sampled CDF distributions in milliseconds up to 5000 ms, even though none of the considered models and parameter sets produced RTs that large. CAF distributions where sampled by 100 intervals.

In this respect, the SPE criterion has a great advantage. It requires only to store the summary statistics (quantiles, error proportions) of the simulated data and not the complete distributions. This decreases the file size of the stored grid by factor 100. Moreover, computation time for the grid search decreased by factor 3. Grid search with SPE took about 1 *min* for a model with seven parameters. However, generating the grid lasted about 3 days, calculated single threaded on a modern prosumer processor, or about one hour, 32 threaded. These costs are, at least partly, compensated by a faster model fits, because the Simplex usually converges much faster starting from the selected grid values than with randomly selected start values. The latter aspect makes grid search especially attractive for fitting data from individuals of a large group, because the same grid can be used for all data fits.

As mentioned, White et al. assessed the general recovery performance of a model for a given sample size by summing their goodness-of-recovery over the populations (*N*_*pop*_ = 100) and model parameters. To have a measure that is independent of these quantities, we used the average performance as measure. Thus, as recovery measure *η* for a model with *N*_*par*_ parameters and a given sample size we used:

$$ \eta =\frac{1}{N_{par}}\sum \limits_{i=1}^{N_{par}}\left(\frac{1}{N_{pop}}\sum \limits_{j=1}^{N_{pop}}\frac{\left(\left| simulated{\theta}_{ij}- recovered{\theta}_{ij}\right|\right)}{range{\theta}_{ij}}\right). $$

## Results

### Replication

Figure 1 shows the recovery performance of the three models in the condition that is similar to that in White et al. (2018), i.e., the goodness-of-fit measure was chi^2^ and the start-value method was rand. As can be seen, there is some variation between the five individual simulations. Interestingly, although recovery improves with sample size, the variation remains almost unchanged. The blue crosses represent the results obtained by White et al. (error data derived from the CAFs). As can be seen, for the SSP and DMC models most of the crosses lie within confidence intervals. For the DSTP model there seems to be a systematic deviation. Except for the smallest sample size, recovery was worse in our study.

Taken together, by and large, we were able to replicate the results of White et al. Although there were some deviations, the relative performance between the three models was the same.

### Effect of fit procedure

To reduce variability for further analyses, the individual samples were combined to form a singly sample of *N*_*pop*_ = 500. Figure 2 shows the mean recovery performance of the models for the different fit procedures and conditions. For analyzing the results, the goodness-of-recovery measures were subjected to a four-way ANOVA with independent factor *model* (DSTP, DMC, and SSP) and the three repeated-measures factors *sample size* (50, 100, 200, 500, 1000, and 5000), *fit criterion* (chi^2^, and SPE), and *start-value method* (rand, and grid search).

The analysis revealed that all main effects were significant (for the *F* and *p* values see Table 1). Concerning the factor *model*, the DMC model recovered best (0.116), followed by the SSP model (0.123) and, with greater distance, the DSTP model (0.181). With respect to *sample size*, recovery improved with an increasing sample size (0.158, 0.143, 0.128, 0.122, 0.111). Further, the fit criterion SPE produced better recovery than chi^2^ (0.135 vs. 0.145). Finally, grid search was a better start-value method than rand (0.126 vs. 0.154).

**Table 1:** Result of the ANOVA. SVM: start-value method, NS: sample size, FitCrit: fit criterion

(Intercept)	*F*(1, 1497) = 26016.46, *p* < .001, petasq = .95
Model	*F*(2, 1497) = 569.34, *p* < .001, petasq = .43
NS	*F*(4.34, 6494.18) = 629.85, *p* < .001, petasq = .30
FitCrit	*F*(1, 1497) = 155.79, *p* < .001, petasq = .09
SVM	*F*(1, 1497) = 1026.58, *p* < .001, petasq = .41
Model × NS	*F*(8.68, 6494.18) = 14.98, *p* < .001, petasq = .02
Model × FitCrit	*F*(2, 1497) = 1.51, *p* = .221, petasq < .01
Model× SVM	*F*(2, 1497) = 118.08, *p* < .001, petasq = .14
NS × FitCrit	*F*(4.77, 7141.41) = 16.28, *p* < .001, petasq = .01
NS × SVM	*F*(5, 7485) = 19.73, *p* < .001, petasq = .01
FitCrit× SVM	*F*(1, 1497) = 0.83, *p* = .362, petasq < .01
Model × NS × FitCrit	*F*(9.54, 7141.41) = 3.77, *p* < .001, petasq < .01
Model × NS × SVM	*F*(10, 7485) = 6.45, *p* < .001, petasq < .01
Model × FitCrit × SVM	*F*(2, 1497) = 7.63, *p* < .001, petasq = .01
NS × FitCrit × SVM	*F*(5, 7485) = 0.43, *p* = .826, petasq < .01
Model × NS × FitCrit × SVM	*F*(10, 7485) = 0.93, *p* = .500, petasq < .01

However, there were also several interactions. The factor *model* interacted with *start-value method*. As can be seen in Fig. 2, although all models benefitted from grid search relative to the rand method (all differences were significant), the DSTP model benefitted more (Δ 0.0462) than the other two models (DCM Δ 0.0222, SSP Δ 0.0150).

*Model* also interacted with *sample size*. However, there was also a significant three-way interaction between *model*, *sample size*, and *start-value method*. As can be seen in Fig. 2, for the rand method, the improvement with sample size was smaller for the DSTP model (from *N*_50_ = 0.226 to *N*_5000_ = 0.190) than for the other models (DCM: 0.175 to 0.091, SSP: 0.165 to 0.105). In contrast, for the grid-search method, the decrease was similar for the models (DSTP: 0.197 to 0.124, DCM: 0.159 to 0.064, SSP: 0.154 to 0.091).

In addition, there was also a three-way interaction between *model*, *start-value method*, and *fit criterion* (see Fig. 2). Further analyses revealed that for the grid-search method the advantage of SPE over chi^2^ (DMC: Δ 0.013, DSTP: Δ 0.009, SSP: Δ 0.011) was significant for all models. For the rand method, however, it was significant only for the DSTP model (Δ 0.015) and the SSP model (Δ 0.010), but not for the DMC model (Δ 0.005).

Finally, there was a significant three-way interaction between *model*, *sample size*, and *fit criterion*. It indicates that the advantage of the SPE over chi^2^ increased with increasing sample size. The increase, however, was different for the models. Further analyses revealed that the two-way interaction between sample-size and fit criterion was significant for DMC model, *F*(4.59, 2289.30) = 6.48, *p* < .001, petasq (partial eta-squared) = .01, and for the SSP model, *F*(4.55, 2272.66) = 13.70, p < .001, petasq = .03, but not for the DSTP model, *F*(4.90, 2445.55) = 1.76, *p* = .120, petasq < .01.

### Recovery of individual parameters

The previous analysis concerned the performance of each model averaged across the parameters. Figure 5 in Appendix A shows the performance for the different conditions and models separately for the individual parameters. As can be seen, recovery performance differs to some extent between the parameters. For the DSTP model, grid search improved recovery for all parameters, whereas for the two other models this was the case only for about half of the parameters.

### Correlation between recovered and original parameters

A further method for assessing recovery performance is to consider the correlation between the recovered parameter values and the original ones. In this connection, White et al. (2018) observed that some parameters were not accurately recovered, because they can trade-off with each other, at least to some extent. For the SSP model, this concerns the parameters *sd*_*a*_ and *r*_*d*_, and for the DSTP model the parameters *μ*_*fl*_ and *μ*_*RSS*_. This problem was resolved by taking the ratio of the respective parameters (i.e., *μ*_*fl*_/*μ*_*RSS*_ and *sd*_*a*_/*r*_*d*_). Here, we also considered these composite parameters.

**Fig. 3:**
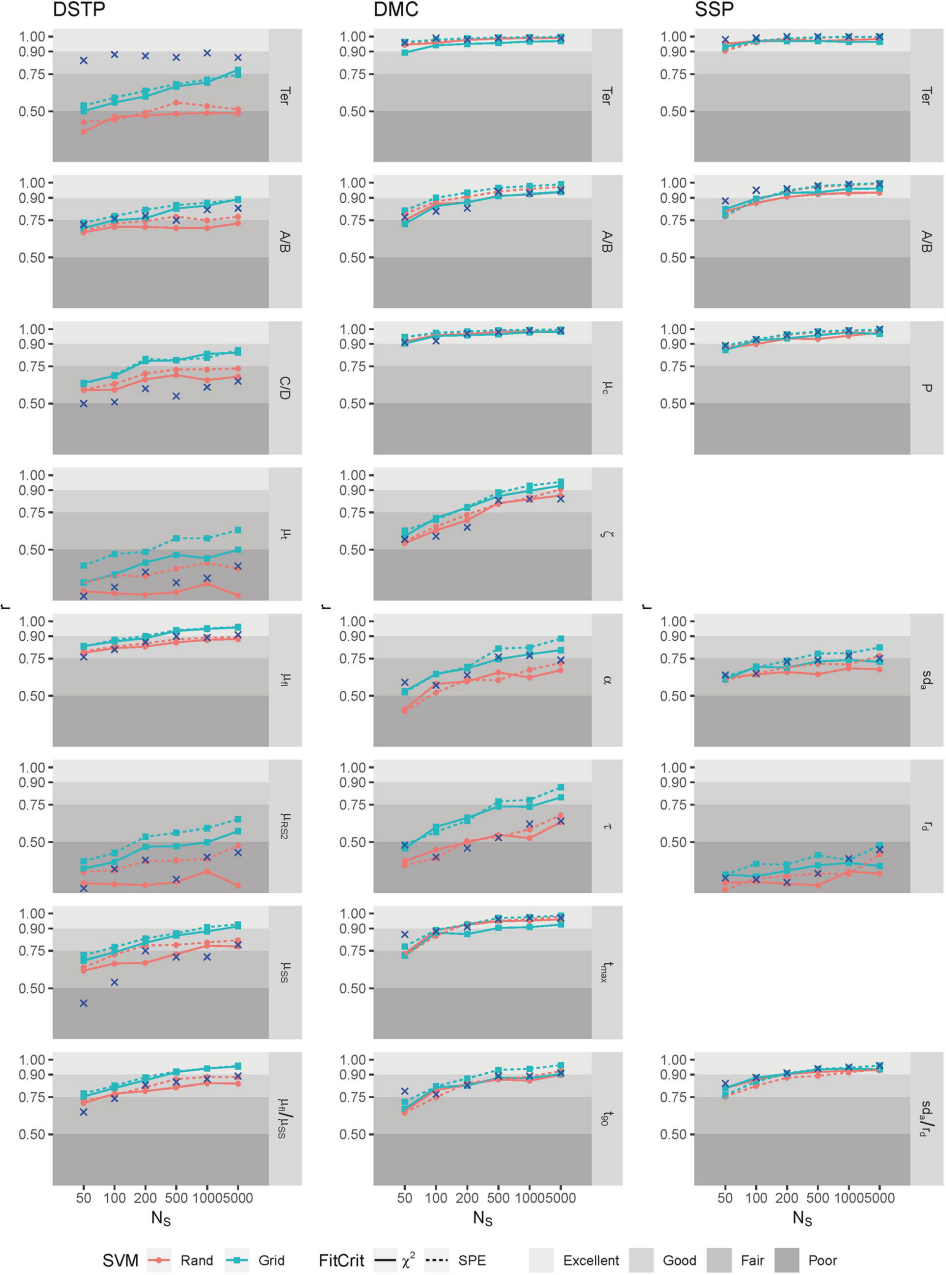
Correlations between the original and recovered parameter values for the three models. The *colored graphs* represent different combinations of start-value condition and fit criteria. The *shaded areas* reflect the evaluation boundaries for the recovery performance after White et al. (2018). The *blue crosses* represent the correlations found in that study

Figure 3 shows the obtained correlations for the parameters^1^ and composites. Like White et al., we categorized each correlation (quality of recovery) *r* as poor, if *r* < .5, as fair if .5 < *r* < .75, as good if .75 < *r* < .9, and as excellent if *r* > .9. In Fig. 3, the corresponding areas are indicated by the gray level of the background. Moreover, the blue crosses show the correlations observed by White et al. The red solid lines (rand, chi^2^) are comparable to the results in that study. As can be seen, for most models and parameters, the correlations are similar to those in White et al. One striking exception is the correlation for *t*_*er*_ of the DSTP model, which is much smaller in the present study.

Overall, the pattern of results is compatible to that in Fig. 5. For most parameters the correlation increased more or less with sample size. Moreover, grid search produced higher correlations, compared to the rand method. This holds especially for the DSTP model. Also, the SPE goodness-of-fit measure led to higher correlations for most parameters and almost never to smaller ones. Especially successful was the combination of grid search and SPE. For the DSTP model, the correlations for parameters *μ*_*RS2*_ and *μ*_*t*_ increased substantially. But also those for parameters *μ*_*SS*_ and *μ*_*fl*_ profited, so that it seems no longer necessary to use their combination. For the SSP model, the correlations for parameter *r*_*d*_ profited most, but still remains in the category “poor” of recovery performance. Therefore, it still makes sense to combine this parameter with *sd*_*a*_. The corresponding composite parameter did not improve by grid search, which, however, is due to a ceiling effect.

### Relation between goodness of fit and parameter recovery

We have seen that the considered fit procedures produced different recovery performance. An interesting question is to what extent this performance was related to the fit performance. Because there were some outliers, we trimmed the goodness-of-fit data. In a first step we discarded very extreme outliers (112), and in a second step all values more than two standard deviations above the mean. In all, 3.88% of the data were discarded. The trimmed means of the chi^2^ and SPE values for the different models and conditions are listed in Tables 2 and 3, respectively. As can be seen, for the DSTP model grid search also led, on average, to a better fit for each sample size than the rand procedure. With one exception, this was also the case for the SSP model. Merely for the DCM model the result was different. Here, grid search improved the fit only in combination with the SPE measure. For chi^2^, there was an improvement only for the largest sample size.

**Fig. 4:**
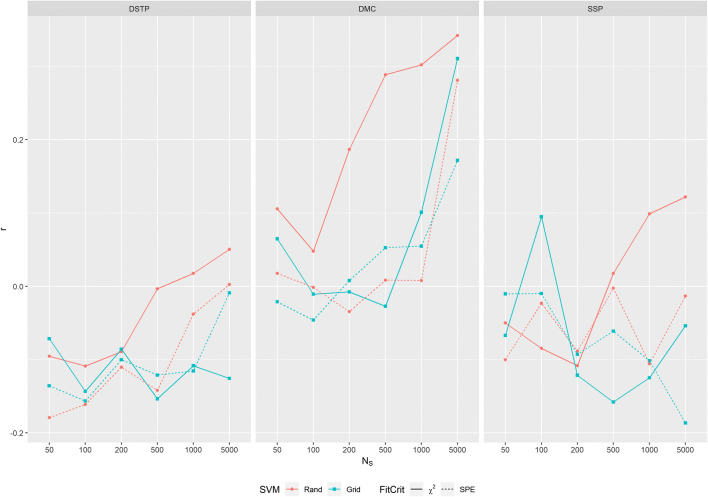
Correlation between goodness of recovery (*η*) and goodness of fit (chi^2^ or SPE) for the different models and fit conditions

**Fig. 5:**
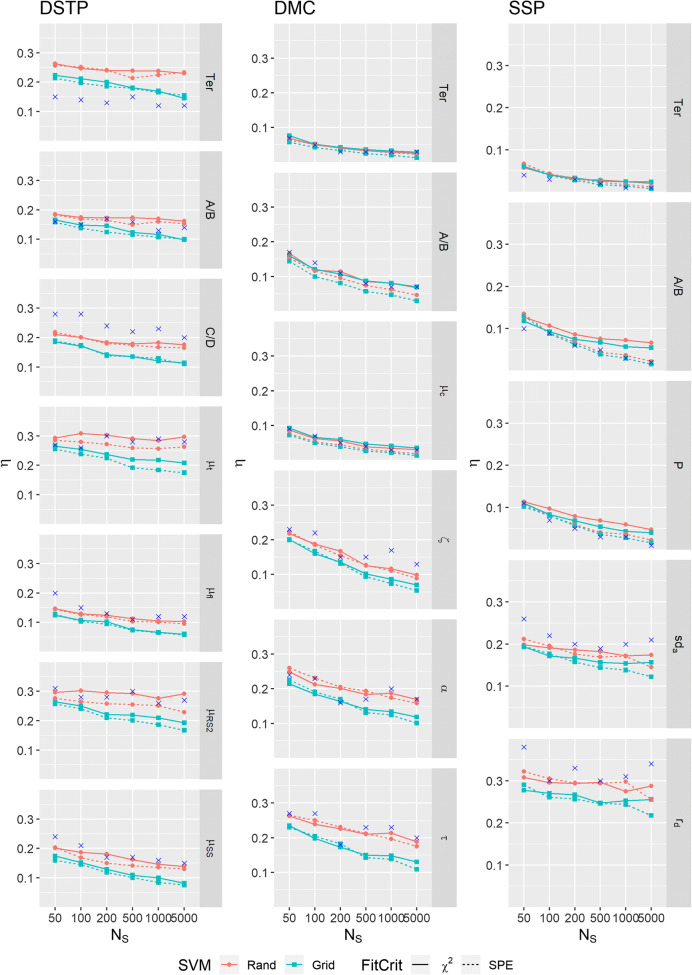
Recovery performance for all parameters regarding the three models. The *colored lines* show the results for different start-value methods (SVM). The line style distinguishes the used fit criterion. *Blue crosses* represent the results from White et al. (2018)

If poor recovery is systematically due to a poor fit, then the two corresponding measures should also be correlated. To test whether this was indeed the case, we correlated the goodness-of-fit values with the corresponding goodness-of-recovery values across the 500 populations for each model and condition. The results are shown in Fig. 4. As can be seen, substantial correlations are practically absent. For the DSTP and SSP models the small correlations are mostly negative and close to zero. For the DMC model they vary closely around zero. Merely for the largest sample size the correlation increased to some extent.

## Discussion

One aim of the present study was to examine whether the results of White et al.’s (2018) parameter-recovery study can be replicated. For this objective we simulated flanker-task data for the three conflict-task models DSTP, DMC, and SSP in the same way as White et al. (2018). In addition, we repeated the simulations four times. Our results show that the replication was by and large successful. One noticeable exception is the DSTP model, for which recovery performance was worse than in White et al.’s study, but mainly for two of the seven model parameters. It should be noted that these conclusions are based on the comparison of White et al.’s data, which comprise 100 populations, with our data, which represent 500 populations. By increasing the number of populations from 100 to 500 we largely reduced the variance of the recovery performance. Had we also simulated only 100 populations, the replication would likely have been worse. This demonstrates that even for 100 populations, there is considerable variability in the data.

In any case, despite some variation, the relations between the different models could be confirmed. Recovery performance was best for the DMC and SSP models and somewhat worse for the DSTP model. Importantly, the difference in recovery performance between the models could be reduced by applying a more sophisticated fit procedure. Improving the fit procedure was the main goal of our study, because parameter recovery strongly depends on the effectivity of this procedure. The task of a fit procedure is to find the set of parameter values that produces the best fit to the observed data. For this objective, fit routines minimize a function that maps parameter values and observed data to a fit criterion (see Eq. 4). The parameters that minimize the fit criterion are then considered as estimate of the population parameters. A problem with applying minimization algorithms is to find the global minimum, because they often get trapped in a local minimum. To circumvent this problem, White et al. (2018) run 20 fit processes for each conditions, each of which started with a different and randomly selected set of parameter values. Twenty start values are more than usually applied in modeling studies. However, as our results show, even this number does not guarantee good parameter recovery.

In the present study, we were able to improve recovery performance by adding a grid-search procedure. That is, instead of relying on a random selection of 20 parameter sets, we systematically chose start values based on a grid search. For this objective, we defined an equally spaced grid as subset extending across the whole parameter space. Then data were simulated for all points in the grid and matched to the to-be-modeled data. The 20 parameter sets that produced the best match were used as start values. As a result, grid search improved recovery performance substantially for all models. However, the improvement was especially pronounced for the DSTP model. Thus, it seems, that the more complex a model is and the more parameters it has, the more it benefits from the grid-search method.

Based on preliminary simulations, we chose a grid size of six. Recovery performance could presumably have been improved by a denser grid. Whether such an effort is worthwhile, however, depends on several conditions such as the model type and the availability of time and/or computational power. Moreover, there is certainly an uncanny valley, because the grid serves to minimize errors of the subsequent simplex method. However, if a grid is extremely dense, then subsequent simplex calculations would be worthless. In the present study we also used the same number of sample points for all parameters in the grid. It might be advantageous, though, to adapt the number of points for each parameter to its range or importance. Thus, the concrete representation of a grid has to be decided from case to case. This decision process should generally be beneficial for DDM modeling, because it leads to a better understanding of reasonable parameter values for a given problem.

In addition to chi^2^, we also applied the SPE (squared percentage error) as goodness-of-fit measure (see Eq. 6). Instead of merely comparing observed and predicted proportions of correct responses related to CDF RTs and of accuracies in CAF intervals, the SPE uses all available data in the summary statistics, which increased the number of values for comparison for each data sample from 20 to 30. This approach not only reduced the storage and computational resources required for the grid search, it also improved recovery performance. This was the case if combined with randomly chosen start values, but in particular, if combined with grid-search, at least for the DMC and DSTP models.

Thus, our results demonstrate that parameter recovery in DDM modeling not only depends on the model, but also on details of the fit procedure. The success of down-hill minimization algorithms such as the Simplex largely depends on the starting values. As we have shown, even running 20 processes with different randomly selected values does not necessarily produce an optimal performance. Models with more parameters and/or a more complex structure might require more sophisticated procedures. Grid search, i.e., quickly sampling the whole parameter space, seems to be a good method. Although pre-computing the grid takes some time, it can be used for multiple fits as long as the model structure remains the same. For instance, the same grid can be used for estimating parameter values for individuals in a group of persons. Because data fitting is not only superior but also much faster with start values selected by grid search, the effort and time spent for computing the grid quickly pays off.

Grid search also improved the goodness of fit, at least for two of the three models. However, it also became clear by our results that the relation between parameter recovery and goodness of fit is not straight forward. To our surprise, there were no systematic correlations between the two criteria. Although it is obvious that a good fit does not guarantee good recovery, we had expected that good recovery produces a good fit, and thereby a moderate correlation. Our results show that this is not the case.

There are at least three possible reasons for the low or absent correlation between fit and recovery. First, the data samples might be a poor representation of the corresponding population, i.e. of ***R*** (see Eq. 1). This is especially likely for a sample of small size, which is usually rather noisy, i.e., ***e*** in Eq. 2 is large. If the fit procedure then estimates parameters for the data sample by simulating a large number of trials, which leads to a small ***e***, then it is highly probable that the obtained parameter set differs from that used for generating the data sample. The larger the difference between sample size trial size, the bigger this problem is. A solution could be to use a larger sample size so that the population is better represented, which should reduce the variance of recovery. However, as our results show, this is not the case. The variance hardly decreases with sample size (see Fig. 1), which could be a result of the still present difference between the *n* in observed and simulated data. Nevertheless, a large sample might be favorable, because it increases the correlation between fit and recovery, even if only to a small extent and mainly for the DSTP and DMC models (see Fig. 4). If increasing the sample size is not possible, then one might fit the model with a trial size similar to the sample size. Whether this really helps, however, has to be shown in later studies.

Second, even if the data samples are good representations of the population, there is the possibility that different parameter sets relate to the same data sample, i.e., different ***p*** map to the same ***r*** (Eq. 1). One measure to prevent this problem is to choose the smallest possible number of parameters so that Eq. 2 approximates a one-to-one mapping. Such an endeavor, however, is counteracted by small *n*, because the resulting large noise produces overlapping solutions spaces for different parameter sets.

Third, it is possible that the fit procedure does not find the optimal parameter set, because the solution space of the fit criterion is rather shallow (Eq. 3), i.e., many parameter sets lead to the same value of *g*. It is important to note that this possibility is different from the previous one. There, we considered the mapping of a multidimensional value ***p*** to another multidimensional value ***r***. Here, we focus on the mapping of two multidimensional values to a scalar (see Eq. 3). To make the occurrence of this third possibility less likely, one might use an extended summary statistic for representing the data sample, because this should make the solution space of the fit criterion more complex and, consequently, less shallow.

Taken together, our study shows that grid search allows to apply a sophisticated fit procedure for conflict DDMs that improves model fitting as well as parameter recovery, relative to current standard procedures. The advantage is the greater the more complex a model is. Accordingly, by applying grid search, the difference between models of different complexity with respect to fit quality and parameter recovery can be reduced. However, our simulations also show that an improved model fit and parameter recovery does not necessarily increase the correlation between these two quality features. Does the observed low correlation mean that grid search is useless? Certainly not. Grid search is still highly recommended. The missing correlation between fit and recovery merely means that the parameter set resulting from the best fit is not necessarily the best representation of the parameters in the population. The obtained representation is nevertheless relatively good, compared to those produced by the standard method. An appropriate strategy for parameter selection after grid search might be to choose the parameter set among the best fitting ones that is most plausible with respect to the situation in which the behavior was observed. It is not unlikely that these parameters represent the population better than the best fitting ones.

### Availability

To make the grid search for different DDMs convenient for researchers, we provide a corresponding R package on GitHub: https://github.com/Pelzer402/DDModeling.git.

## Figures and Tables

**Fig. 6:**
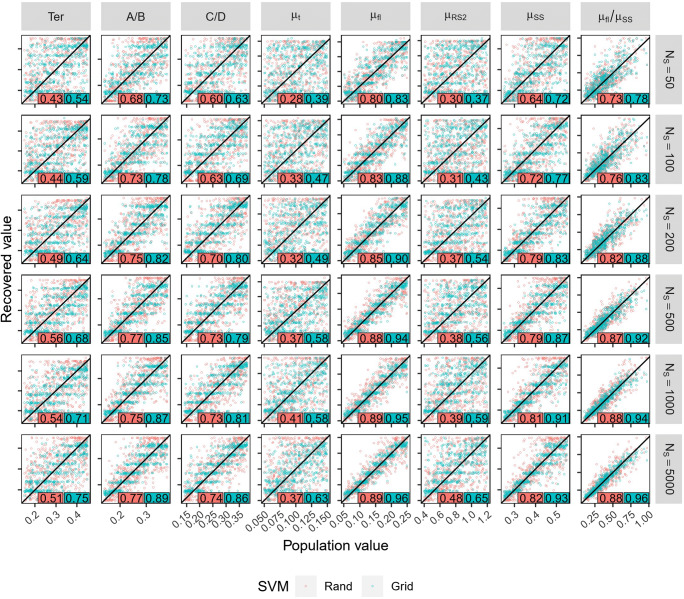
Recovery performance of DSTP model for the individual parameters and the two start-value methods (SVM). Fit criterion was SPE. Simulated population values are plotted against the recovered values for each parameter. Values along the diagonal indicate good recovery. See also the corresponding correlations in the colored boxes

**Fig. 7:**
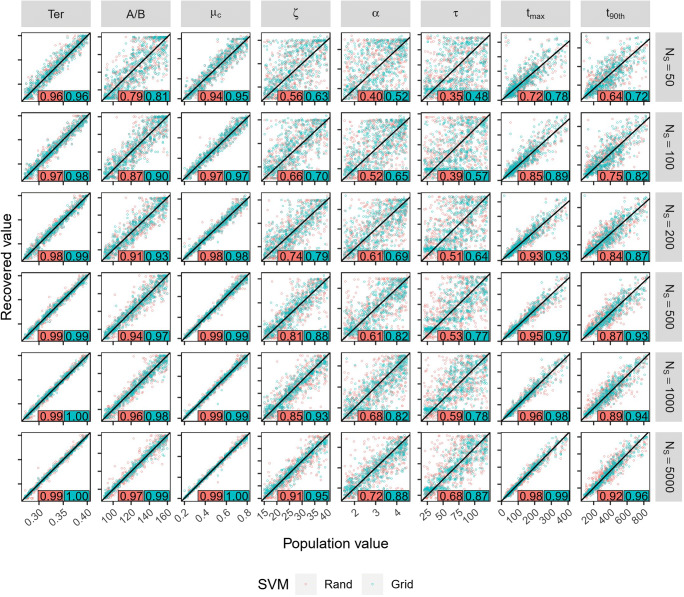
Recovery performance of DMC model for the individual parameters and the two start-value methods (SVM). Fit criterion was SPE. Simulated population values are plotted against the recovered values for each parameter. Values along the diagonal indicate good recovery. See also the corresponding correlations in the colored boxes

**Fig. 8:**
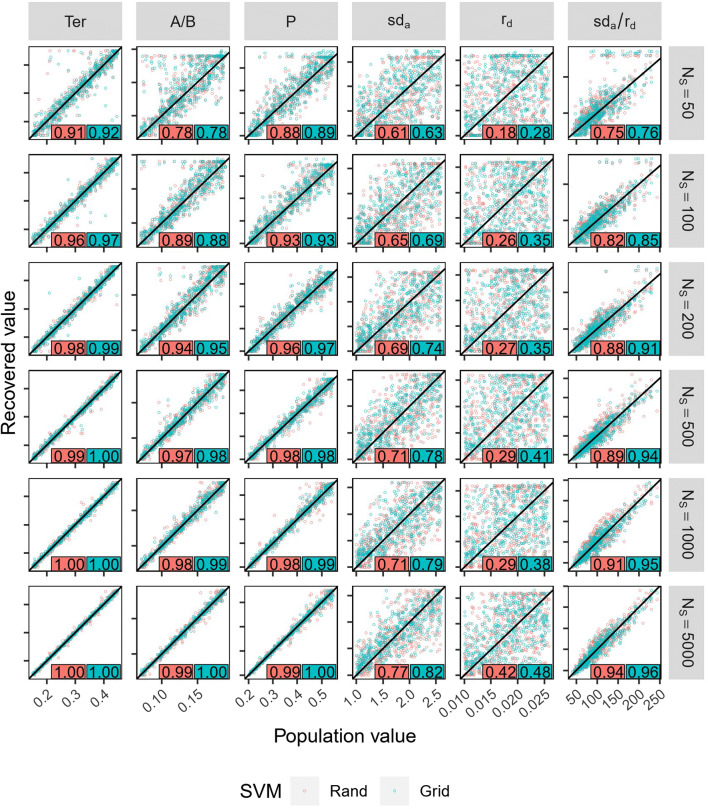
Recovery performance of SSP model for the individual parameters and the two start-value methods (SVM). Fit criterion was SPE. Simulated population values are plotted against the recovered values for each parameter. Values along the diagonal indicate good recovery. See also the corresponding correlations in the colored boxes

## Appendix A

**Table 2.** Chi^2^ goodness-of-fit value averaged across the trimmed data of the 500 populations. The *values in parentheses* are the number of values out of 500 that were used for calculating the average

	DSTP		DMC		SSP	
	Rand	Grid	Rand	Grid	Rand	Grid
50	8.11 (497)	6.94 (477)	4.57 (488)	7.88 (479)	9.13 (470)	9.20 (469)
100	7.55 (499)	6.06 (468)	4.87 (490)	7.25 (443)	9.09 (471)	7.66 (473)
200	7.22 (475)	6.07 (475)	5.43 (493)	7.16 (462)	9.41 (477)	8.30 (476)
500	9.05 (486)	6.16 (482)	6.34 (497)	8.42 (474)	10.3 (467)	8.43 (474)
1000	11.6 (484)	6.59 (480)	9.15 (498)	9.90 (480)	12.7 (476)	9.62 (471)
5000	34.5 (484)	9.61 (483)	29.0 (496)	22.9 (481)	30.5 (479)	14.0 (484)

**Table 3.** SPE goodness-of-fit values averaged across the trimmed data of the 500 populations. The *values in parentheses* are the number of values out of 500 that were used for calculating the average

	DSTP		DMC		SSP	
	Rand	Grid	Rand	Grid	Rand	Grid
50	0.0174 (482)	0.0157 (482)	0.0328 (470)	0.0313 (469)	0.0433 (449)	0.0431 (447)
100	0.0090 (483)	0.0076 (481)	0.0170 (471)	0.0161 (473)	0.0235 (478)	0.0226 (474)
200	0.0054 (489)	0.0042 (490)	0.0084 (477)	0.0076 (476)	0.0132 (484)	0.0125 (483)
500	0.0032 (492)	0.0019 (491)	0.0038 (467)	0.0031 (474)	0.0065 (490)	0.0054 (488)
1000	0.0020 (486)	0.0010 (486)	0.0023 (476)	0.0016 (471)	0.0032 (485)	0.0029 (491)
5000	0.0016 (490)	0.0005 (486)	0.0011 (479)	0.0006 (484)	0.0011 (485)	0.0006 (485)

## Footnotes

1 Scatterplots corresponding to the individual correlations are shown in Figures A2 and A3 in the Appendix
